# Impact of ecological presence in virtual reality tourism on enhancing tourists’ environmentally responsible behavior

**DOI:** 10.1038/s41598-024-56615-z

**Published:** 2024-03-11

**Authors:** Zhen Su, Biman Lei, Dandan Lu, Shuchen Lai, Xijing Zhang

**Affiliations:** 1 Department of Tourism Management, Business School, Guangxi University, No. 100 East Daxue Road, Nanning, 530004 China; 2 Department of Tourism Management, Business School, Guangxi University of Finance and Economics, Nanning, 530008 China; 3 Guangxi Development Strategy Institute, Nanning, 530004 China; 4 Key Laboratory of Interdisciplinary Science of Statistics and Management, Nanning, 530004 China

**Keywords:** Virtual reality tourism, Tourists' environmentally responsible behavior, Ecological presence, Biospheric values, Environmental self-identity, Personal norms, Psychology and behaviour, Sustainability

## Abstract

In an era where environmental conservation is increasingly critical, identifying pathways through which technological innovations like virtual reality tourism (VRT) can promote sustainable behaviors is vital. This study investigates the impact of ‘ecological presence’, a newly proposed sub-dimension of presence in VRT, on tourists’ environmentally responsible behavior (TERB). Through structural equation modeling and fuzzy set qualitative comparative analysis of data from 290 participants, we unveil that ecological presence—defined as the authenticity and immersion of tourists in virtual ecological environments—significantly bolsters biospheric values, environmental self-identity, and personal norms. Additionally, our findings indicate that ecological presence in VRT indirectly promotes TERB, predominantly through the mediation of enhanced biospheric values and environmental self-identity. Notably, ecological presence, biospheric values, and environmental self-identity constitutes a sufficient condition for achieving a high level of TERB. This research highlights the potential of VRT as an innovative tool for tourism administrators to foster environmental stewardship, offering a novel approach to leveraging technology for conservation efforts.

## Introduction

In light of the pressing concerns surrounding climate change and environmental degradation at many tourist destinations, understanding how to foster tourists' environmentally responsible behavior (TERB) has become pivotal^1–3^. Countries are adopting innovative strategies to encourage this behavior: Norway promotes the “Leave No Trace” principle to preserve its natural beauty through strict regulations^4^, and Palau requires visitors to sign the “Palau Pledge”, an eco-pledge committing them to environmentally responsible behavior during their stay^5^. Prior research has examined the impact of traditional tourism on TERB, which encompasses tourists' commitment to environmentally beneficial practices^6^, underscores the significance of environmental self-efficacy and self-image in fostering ecological consciousness^1,7^. Amidst the COVID-19 pandemic, the rise of virtual reality (VR) technology has introduced virtual reality tourism (VRT), a novel concept that uses 3D technology to create immersive travel experiences with visual, motion, and audio cues^8–10^. This serves as an innovative alternative to traditional tourism, providing enriching experiences that have the potential to positively influence tourists’ behaviors towards the environment^11,12^. The application of VRT as an educational and awareness-building tool offers a promising avenue to instill sustainable practices in tourists, thereby potentially minimizing the physical impact on sensitive environments^13,14^.

In examining VRT's role in heightening environmental consciousness and advancing sustainable practices, the current body of literature can be categorized into two main areas. The first area investigates the capacity of VRT to heighten individuals' willingness to engage in environmental conservation by delivering immersive experiences of nature. This includes exposure to awe-inspiring virtual landscapes^15^, simulated glacier tours^16^, and virtual deep-sea adventures^17^. These studies provide evidence that VRT can effectively amplify individuals’ intentions toward environmental preservation, cultivating favorable attitudes and proactive behaviors toward safeguarding our planet. The second area of research considers VRT as an innovative form of sustainable tourism in the post-pandemic era, examining how virtual experiences can encourage pro-environmental behaviors^11^. This research highlights that concerns about travel and positive perceptions of VRT are motivating a shift towards virtual tourism, offering a potential solution to lessen the demand for physical travel and enhance pro-environmental intentions^11^. While the influence of VRT on TERB has been recognized, existing studies fall short of comprehensively analyzing how an ecological environment-related presence in VRT settings impacts TERB. This gap underscores the necessity to delve into the specific ways in which ecological immersion within VRT environments drives the mechanisms underlying TERB.

Exploring the impact mechanism of this specific presence on TERB is an important part of understanding the environmental effects of VR. Presence, defined as the mental state where a user feels fully immersed in a mediated environment^18^, is a pivotal concept for understanding VR's effectiveness in its intended context^19^. This state of presence, achieved through VR systems' image display, audio synchronization, tactile feedback, and user movement tracking, bridges the physical and virtual realms, thereby enriching user engagement and perception within simulated environments^20^. In this study, “ecological presence” is defined as the authenticity and immersion of users in VR who feel the ecological environment and the ecological connections and interactions between various internal elements. It adds an ecological dimension to the traditional sense of presence. Additionally, the values-identity-personal norms (VIP) model, which outlines the causal relationships among biospheric values, environmental self-identity, and personal norms, is a vital framework for explaining various environmental behaviors^21–23^. In the immersive environments of VRT, consumers may experience a sense of awe that is distinct from traditional travel^15,24^. Ecological presence is not only a state but also a causal factor that may influence cognitive variables^19^ and, consequently, could modify the established understanding of how biospheric values, environmental self-identity, and personal norms contribute to TERB within virtual environment.

This study investigates the impact of ecological presence in VRT on TERB at China's Jiuzhaigou scenic spot, utilizing Cognitive Appraisal Theory (CAT) and the VIP model to examine how virtual ecological presence impacts biospheric values, environmental self-identity, and personal norms. Employing Structural Equation Modeling (SEM) and Fuzzy Set Qualitative Comparative Analysis (fsQCA), we analyze the data to decipher these mechanisms. The anticipated contributions of this research are threefold: (1) By introducing ecological presence as a new sub-dimension based on the presence experienced within VRT, this study offers a novel perspective for examining the environmental impact of VRT, enriching the conceptual framework of presence in virtual environments. (2) This research advances theoretical insights by demonstrating how VRT's immersive experiences activate biospheric values, environmental self-identity, and personal norms, effectively bridging the gap between VR ecological presence and environmental stewardship through the application of CAT. (3) Furthermore, the study provides a methodological framework for future research by identifying conditions that promote high levels of TERB, thereby deepening our understanding of the relationship between digital immersion and environmental conservation initiatives.

## Literature review and development of hypotheses

### Presence and ecological presence

In the realm of VR, presence is considered instrumental in driving user attitudes and behaviors toward virtual environments^19^. The immersive experience that comes with a sense of presence utilizes the human sensory experience to make individuals feel like they are in the real world^25^. Previous studies have connected the sense of presence with technological advancements, particularly the “perceived presence in computer-mediated environments”^26^. Park et al. conceptualized presence as “a mental state in which the virtual nature of the experience goes unnoticed”^25^. Presence has been used in several areas of research, including medical clinical treatment^27^, sustainable consumption^28^, games^25,29^ and education^30^.

With burgeoning interest in VRT, presence has also been incorporated into it^18,19^. In tourism, presence has been primarily employed to examine tourists’ attitudes and behaviors^31^, such as their acceptance of VRT, experiences, and satisfaction^32^, as well as their motivations and intentions to visit destinations^19^. Presence occupies a critical position in VR technology, as it enhances VR authenticity, thereby improving the user experience^33^. Various VR devices, such as fully immersive devices, semi-immersive devices, 360° interactive VR videos^34^, engender varying degrees of presence, subsequently influencing tourists’ perceptions in diverse ways^26^.

Advancing from the established research on presence in VR and VRT environments, this study pioneers the notion of 'Ecological Presence' within the VRT context. This concept captures the unique sense of immersion and realism experienced by users when interacting with ecologically themed virtual environments, underscoring the connections and interactivity between various ecological components^12,15^. Ecological presence builds upon the traditional sense of spatial immersion by incorporating a directed focus on the ecological environment, where users are not only present within a digitally recreated space but are actively engaged with its ecological attributes. It represents a specialized facet of presence that could reshape the design and application of VRT experiences, aiming to enhance ecological awareness and promote sustainability within the burgeoning field of VRT.

### Cognitive appraisal theory

Foroughi et al. highlighted that the CAT can be employed to clarify the connection between emotions and experiences^35^. The key point of CAT is that specific emotional reactions are tied to particular referents, resulting in corresponding behavioral consequences^36^. While emotions are not directly determined by external stimuli or events, individuals' interpretations and evaluations of stimuli and events affect their emotional responses^37^.

CAT posits that experiential evaluations stem from evaluative dimensions, eliciting specific emotions, subsequently influencing behavioral responses^36^. So far, scholars commonly employ CAT to expound on pro-environmental behaviors rooted in cognition and emotion^35^. In the realm of VRT, “presence” acts as a referent that can bring about positive evaluations of virtual technology for individuals, subsequently triggering positive emotions^18^. These positive emotions influence specific cognitions and behaviors within the realm of environmental protection.

### Ecological presence and TERB

Ecological presence, while rooted in the physical sense of authenticity or realness within a given space^25,38^, is a nuanced feeling of being connected with the ecological aspects of that environment. In tourism studies, perceived ecological presence during a VR experience can evoke positive emotional experiences specific to the natural world in tourists^16^. CAT suggests that these particular emotional responses, related to ecological referents, lead to differentiated behavioral outcomes^35^. Although external stimuli do not directly elicit emotions, they provide the context for the cognitive evaluation of emotion generation, serving as vital sources of information for individual emotional responses^39^. When immersed in an environment with strong ecological presence, visitors experience the setting as a genuine ecological space, triggering positive emotions^40^. It is posited that a high level of ecological presence during a virtual nature experience may trigger the emotion of awe, a powerful self-transcendent positive emotion that is especially relevant to ecological settings^24,40^. Such a heightened emotional state can influence visitor loyalty and satisfaction^41^and is known to increase prosocial behavior^42^, such as environmental stewardship. In the realm of tourism, TERB is a significant form of prosocial behavior that aligns closely with the concept of ecological presence^43^. Following this theoretical backdrop, the hypothesis is proposed:

H1: Ecological presence positively influences TERB.

### Personal norms

Personal norms are individuals’ moral obligations or duties aligned with their core beliefs^44,45^. These norms considerably influence the realization of a person’s intentions, which are shaped by their perceived obligations, and subsequently affect the probability of corresponding actions^23^. Personal norms are underpinned by self-expectations, sanctions, and obligations, which are fundamentally entrenched in intrinsic values. These norms are formed based on an evaluation of moral obligation or self-awareness pertaining to a particular behavior^46^. Drawing on CAT, a sense of presence in virtual technology can stimulate positive self-transcendent emotions (such as awe)^12,47^, which further influence personal norms^48^. The application of personal norms as a predictive measure for environmentally responsible behavior is widely recognized^1,45,49^. It effectively influence visitors to participate in various behaviors, including garbage cleanup at national parks^44^, reducing waste production, promoting waste recycling during trips^46^, and choosing environmentally friendly options such as cycling^50^. Therefore, the following hypotheses were proposed:

H2a: Ecological presence positively impacts tourists' personal norms.

H2b: Personal norms positively affect TERB.

H2c: Personal norms mediate the relationship between ecological presence and TERB, implying that ecological presence amplifies tourists' personal norms, enhancing TERB.

### Environmental self-identity

Self-identity encapsulates an individual’s self-perception^51^. The formation of self-identity leads to the development of a social identity that affects environmental engagement and actions^21^. Once established, a specific self-identity promotes corresponding behaviors^52^. Sociology and psychology argue that an individual’s self-identity significantly influences their behavior^53^. Environmental self-identity is characterized by the degree to which individuals perceive themselves as environmentalists. In VRT, the immersive environment is capable of simulating a spatiotemporal interweaving of experiences within natural settings^15^, which are seldom encountered in daily life or traditional tourism. This unique characteristic of VRT facilitates the elicitation of awe towards nature more readily^24^. Drawing on CAT, such awe is identified as a catalyst for fostering positive evaluations and emotions concerning environmental protection^15^, subsequently influencing an individual's environmental self-identity^54^. Stronger environmental self-identity corresponds to a heightened belief in environmental protection, increasing the likelihood of exhibiting environmentally protective behaviors^22,51^, such as adopting new energy sources or choosing to cycle^50,53^. Therefore, the following hypotheses were proposed:

H3a: Ecological presence positively influences environmental self-identity.

H3b: Environmental self-identity positively impacts TERB.

H3c: Environmental self-identity mediates the relationship between ecological presence and TERB, implying that ecological presence augments tourists' environmental self-identity, enhancing TERB.

### Biospheric values

Values serve as guiding principles, helping individuals make choices or evaluations about people, behaviors, and events and significantly influencing their attitudes and behaviors^55^. The values activated in a given situation guide an individual’s choice, determining which values are most relevant^56^. According to the values-beliefs-norms (VBN) theory, values influence our environmental worldview (i.e., general beliefs); when environmental changes threaten our perceptions of what we value, beliefs are affected, which affects the norms for taking action^21^. According to CAT, presence in VR technologies can trigger the positive emotions of self-transcendence, especially in virtual natural landscapes^24^. Indeed, such self-transcendent emotions can predict greater personal concern for the biosphere^57^, promoting the emergence of biospheric values. Individuals with biospheric values may commitment to engage in environmentally responsible behavior to protect the biosphere^1^.Therefore, the following hypotheses were proposed:

H4a: Ecological presence positively affects tourists’ biospheric values.

H4b: Biospheric values positively affect TERB.

H4c: Biospheric values mediate the relationship between ecological presence and TERB; that is, ecological presence enhances tourists’ biospheric values, which enhances TERB.

**Figure 1:**
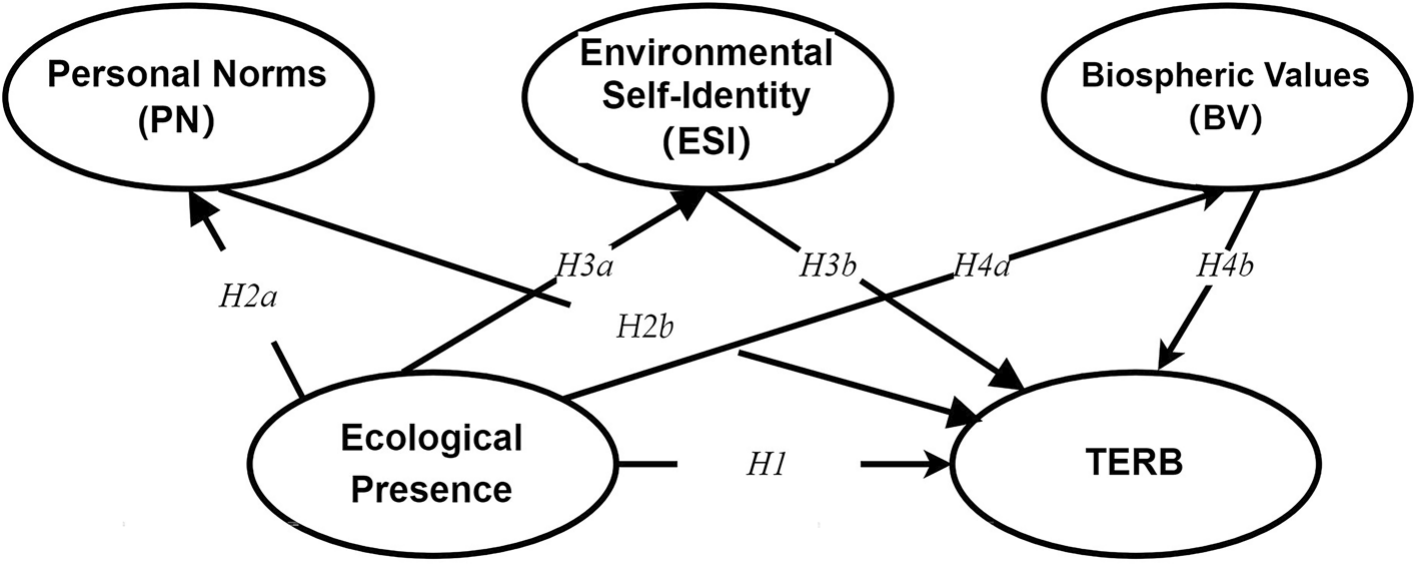
Research model. PN: Personal Norms, ESI: Environmental Self-Identity, BV: Biospheric Values.

Based on these hypotheses, the following research model was constructed, see Fig. 1.

## Methodology

### Experimental design and data collection

We constructed an experiment and invited volunteers to engage in a VRT experience through random sampling at RED, Weibo. Academic Committee of Guangxi University Business School approved the study (No. 2023032201). We strictly follow the Declaration of Helsinki and all research were conducted in accordance with the relevant guidelines and regulations. Before beginning the experiment, each participant gained their informed consent. And informed consent for participation in the research was obtained from the parents and legal guardians of all minor participants. For illiterate participants, informed consent was also obtained from their guardians and legally authorized representatives. All participants were informed that the research purpose, and be told that research team would take anonymous surveys, and the results would only be used for scientific research without revealing personal information. They were asked: “Would you like to participate in this survey?” Those who agreed were invited to participate in the VRT experience.

**Figure 2:**
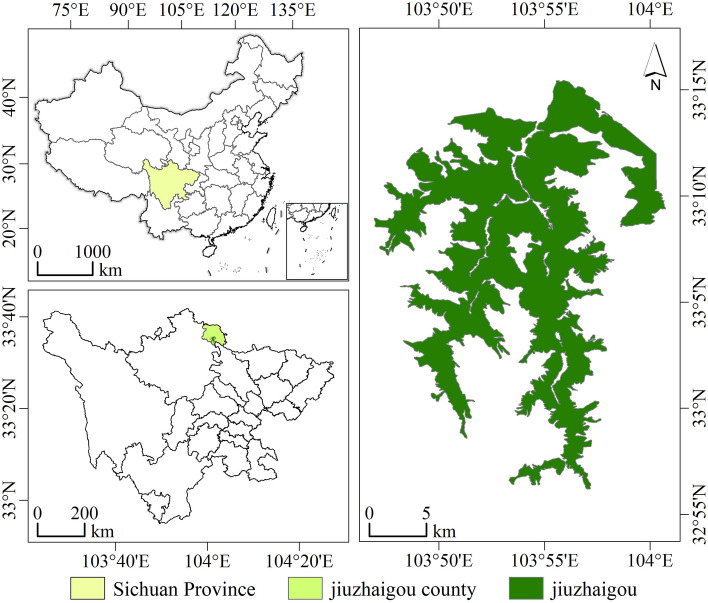
Research area.

The focal point of this case study was Jiuzhaigou, an AAAAA-rated scenic location in China. This unique site is the only tourist destination in China that holds the dual titles of a World Natural Heritage site and World Biosphere Reserve (Fig. 2). Moreover, it is distinguished as the premier nature reserve in China that aims to conserve natural scenery. The Quanjingke Virtual Tourism Network (http://www.quanjingke.com/) crafted a VRT 360° interactive video showcasing the visually stunning various landscapes of Jiuzhaigou. The participants were provided relevant background narratives and could access various scenic locations in Jiuzhaigou. The entire experience lasted approximately 50 s, after which the participants were required to complete a questionnaire.

The questionnaire design selected 7-point Likert scale (“1” = not at all agree and “7” = strongly agree), drew on previous research and encompassed measures from the dimensions of ecological presence, personal norms, environmental self-identity, biospheric values, and TERB. 4-item measured ecological presence^26^, 5-item measured personal norms^1^, 3-item measured environmental self-identity^1,22^,4-item measured biospheric values^22^, environment-saving behavior, environment-promoting behavior, and environment-abiding behavior, total 15-item measured TERB^58–61^ (Supplementary Table S1).

To ensure the reliability of the survey, a pretest was conducted from March 23, 2023 to March 25, 2023. We distributed 93 questionnaires to confirm the validity and reliability of the content^62^. Due to the potentially excessive number of parameters required for estimation, model stability might be compromised. Consequently, before conducting in-depth data analysis, we computed the mean values for the three dimensions of environment-saving behavior, environment-promoting behavior, and environment-abiding behavior ^63,64^. We utilized means as metrics^64^.

The results showed Cronbach’s α for each latent variable ranged from 0.89 to 0.96, and the overall Cronbach’s α was 0.96, surpassing the critical threshold of 0.7. Demographic variables, such as age, gender, and education level, were controlled for to mitigate the potential impact of these variables on the results. Furthermore, to control for the potential influence of the VR context on the study results, participants were asked whether they had previously experienced VR^65^. The online questionnaire was distributed from March 28, 2023 to March 30, 2023, using Questionnaire Star (https://www.wjx.cn/), which is a third-party sample service platform. A total of 331 samples were collected. After excluding invalid questionnaires, 290 participants were included in the final sample. The demographic characteristics of the participants are shown in Table 1. SEM and fsQCA were conducted to assess the causal relationships between variables.

**Table 1:** Descriptive characteristics of the participants.

Demographic Characteristics	Description	Count	Percentage (%)
Gender	Male	104	35.9
	Female	186	64.1
Age	Under 20 years old	7	2.4
	20–29 years old	121	41.7
	30–39 years old	118	40.7
	40–49 years old	29	10
	50–59 years old	13	4.5
	Above 60 years old	2	0.7
Level of Education	Undergraduate and below	54	18.6
	Bachelor	216	74.5
	Postgraduate	11	3.8
	Master/PhD	9	3.1
VR experience	Yes	219	75.5
	No	71	24.5

### FsQCA and calibration

FsQCA examines multi-cause combinations of generated results to facilitate the assessment of nonlinearity, asymmetry, equilibrium, multiple outcomes, and cascading causality^66^. It is a robust tool for identify multiple combinations of conditions about the same result^67^ and compensates for certain shortcomings associated with data processing in multiple regression analyses^68^. Unlike SEM, which focuses on the linear relationship between independent and dependent variables, fsQCA allows for the simultaneous causation inherent in complex multifactorial situations^69^. The combined application of symmetric SEM and asymmetric fsQCA has become commonplace in tourism and hospitality research^67,70^. This combination compensates for the lack of an estimation technique in SEM and the effect of the indicator measurement error in fsQCA to account for complex models^71,72^.

FsQCA necessitates the preliminary calibration of all the data^73^. Consequently, Likert-scale data must be converted into fuzzy membership scores to indicate the degree and extent to which a case belongs to a specific set^74^. This calibration process involves specifying three anchor points: 5% (full non-membership), 95% (full membership), and 50% (crossover point)^75^, thereby transforming raw data into fuzzy sets between 0 and 1^69,76^. Although it is rare for causal conditions or combinations thereof to always adhere to a necessity or sufficiency relationship, it is crucial to assess the extent to which the cases in a dataset satisfy such a relationship. In fsQCA, consistency and coverage provide evaluative evidence^77^. The methodology also requires setting consistency thresholds^71^, proportional reduction in inconsistency (PRI) score thresholds, and case frequency score thresholds^71^, with the consistency threshold ranging from 0 to 1^78^.

FsQCA constructs truth tables and performs sufficiency condition analyses^78^. Subsequently, fsQCA generates three distinct solution terms: complex, parsimonious, and intermediate^76^. Intermediate solution is selected to obtain the final results^78^.

### Ethics declarations

The research involving human participants were reviewed and approved by the Academic Committee of Guangxi University Business School (No. 2023032201). The participants provided informed consent to participate in this study. Informed consent in the research was obtained from the parents and legal guardians of all minor participants. For illiterate participants, informed consent was also obtained from their guardians and legally authorized representatives.

## Results

### Measurement model testing and structural model testing

The measurement model was assessed by reliability analysis, convergent validity and discriminant validity. Initially, a reliability analysis of all items was implemented. Cronbach’s α for each latent variable ranged from 0.76 to 0.85, and the overall Cronbach’s α was 0.91, surpassing the critical threshold of 0.7. All factor loadings exceeded 0.5, thereby reaching acceptable reliability standards^79^. The composite reliability was then assessed, yielding values between 0.77 and 0.85, further indicating the high reliability of our scale^80^. The average variance extracted (AVE) for each latent variable ranged from 0.46 to 0.66. AVE values below 0.5 indicate acceptable results provided that the standardized factor loadings are greater than 0.6^81^. Therefore, our model demonstrated good convergent validity (Table 2). Subsequently, discriminant validity was measured using the model comparison method^82^. As shown in Table 3, the five-factor model demonstrated superior fit with the actual data compared to the other five models (χ^2^ = 335; df = 142; χ^2^/df = 2.37; NFI = 0.88; CFI = 0.93; RMSEA = 0.07). These results indicated that the scales employed exhibited satisfactory discriminant validity.

**Table 2:** Convergence validity test.

Construct	Factor loadings (> 0.7)	Cronbach's α	C.R. (> 0.7)
Sence of ecological presence		0.77	0.77
E-pres1	0.83		
E-pres2	0.80		
E-pres3	0.78		
E-pres4	0.71		
Personal norms		0.84	0.77
PN1	0.82		
PN2	0.80		
PN3	0.79		
PN4	0.79		
PN5	0.75		
Environmental self-identify		0.76	0.85
ESI1	0.76		
ESI2	0.85		
ESI3	0.85		
Biospheric values		0.77	0.77
BV1	0.78		
BV2	0.77		
BV3	0.78		
BV4	0.74		
TERB		0.85	0.85
Environment-saving behavior	0.91		
Environment-promoting behavior	0.88		
Environment-abiding behavior	0.85

**Table 3:** Differentiation validity test ***(p<0.001).

Model	Descriptions	X^2^	df	ΔX^2^	RMSEA	NFI	CFI
Model 1	Five factors: ecological presence, BV, ESI, PN, TERB	335	142		0.07	0.88	0.93
Model 2	Four factors, ecological presence and PN were combined into one factor	461.6	146	126.57***	0.09	0.83	0.88
Model 3	Four factors, ESI and PN were combined into one factor	800	146	239.41***	0.1	0.81	0.84
Model 4	Four factors, BV and ESI were combined into one factor	488.9	146	212.1***	0.09	0.87	0.9
Model 5	Four factors, BV and TERB were combined into one factor	426.9	146	62***	0.08	0.88	0.92
Model 6	One factor: ecological presence, BV, ESI, PN, TERB were combined into one factor	1167	152	740.1***	0.14	0.68	0.71

Before testing the hypotheses, we assessed the goodness of fit of the structural model^80^. The model goodness-of-fit indices (χ^2^ = 357, df = 145, χ^2^/df = 2.64, GFI = 0.88, AGFI = 0.84, CFI = 0.92, and RMSEA = 0.07) indicated that certain criteria were not met. Therefore, to enhance the model’s goodness of fit, the Bollen-Stine bootstrap method was used for model fit adjustment and parameter estimation^83^. The resulting modifications are illustrated in Table 4. The adjusted model exhibited an acceptable goodness of fit^80^.

**Table 4:** Goodness of fit evaluation indexes for structural model.

Index	Value measured	Cut-off value	Decision
CFI	1	> 0.9	Acceptable
GFI	0.95	> 0.9	Acceptable
NFI	0.95	> 0.9	Acceptable
TLI	1	> 0.75	Acceptable
SRMR	0.07	< 0.08	Acceptable
RMSEA	0.01	< 0.06	Acceptable
CMIN/DF	1.03	< 5.0	Acceptable

**Figure 3:**
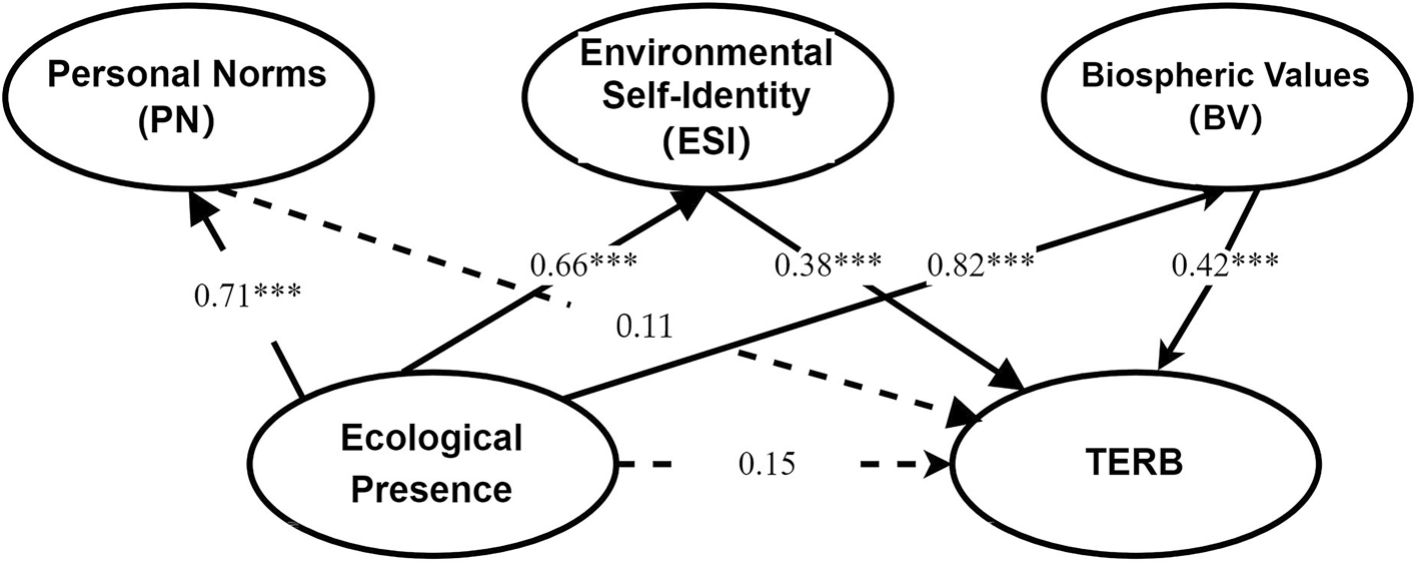
Model measuring.

Then, we began to test the hypotheses by measuring the direct and indirect relationships between the variables and estimating the path coefficients. The results are presented in Fig. 3 and Tables 5 and 6. Ecological presence did not significantly affect TERB (β = 0.15, p > 0.001) but positively influenced personal norms (β = 0.71, p < 0.001). Personal norms did not significantly affect TERB (β = 0.11, p > 0.001), whereas ecological presence positively influenced both biospheric values (β = 0.82, p < 0.001) and environmental self-identity (β = 0.66, p < 0.001). Biospheric values (β = 0.42, p < 0.001) and environmental self-identity (β = 0.38, p < 0.001) positively influenced TERB. These findings supported H2a, H3a, H3b, H4a, and H4b; however, H1 and H2b were not supported.

**Table 5:** Hypotheses testing ***(p<0.001).

Hypotheses	Relationships	β	S.E	C.R	P	Decision
H1	Ecological presence → TERB	0.15	0.13	1.04	0.30	Not supported
H2a	Ecological presence → PN	0.71	0.10	8.90	***	Supported
H2b	PN → TERB	0.11	0.06	1.58	0.12	Not supported
H3a	Ecological presence → ESI	0.66	0.08	7.74	***	Supported
H3b	ESI → TERB	0.38	0.07	5.14	***	Supported
H4a	Ecological presence → BV	0.82	0.09	9.48	***	Supported
H4b	BV → TERB	0.42	0.10	3.82	***	Supported

**Table 6:** Mediation effect analysis.

				Bootstrapping			
SIE	Point estimate	Product of coefficients		Bias-corrected 95%CI		Percentile 95%CI	
		SE	Z	Lower	Upper	Lower	Upper
PN	0.070	0.060	1.110	−0.040	0.200	−0.040	0.200
ESI	0.220	0.070	3.100	0.100	0.390	0.090	0.380
BV	0.310	0.130	2.330	0.070	0.580	0.100	0.620
PN vs. ESI	−0.150	0.010	−1.710	−0.340	0.020	−0.340	0.030
ESI vs. BV	−0.090	0.170	−0.530	−0.380	0.210	−0.440	0.190
BV vs. PN	0.240	0.140	0.002	−0.050	0.490	−0.004	0.540

To further validate our findings, we proposed hypotheses H2c, H3c and H4c to test the mediating effects. The bootstrap method was used to examine the mediating roles of personal norms, biospheric values, and environmental self-identity. Bootstrap 95% CI values showed that biosphere values and environmental self-identity fully mediated the influence of ecological presence on TERB. The values also suggested that there is no mediation effect for personal norms in the influence of ecological presence on TERB. In addition, bootstrap 95% CI values the differences between environmental self-identity and biospheric values indicated that the mediation differences were insignificant. Therefore, the results supported hypotheses H3c and H4c but not H2c. The result showed that ecological presence cannot directly affect TERB, but needs to be affected through two intermediary variables, biospheric values and environmental self-identity.

### Configuration analysis of TERB

To investigate the interplay between independent variables and mediating variables within SEM in fostering a high level of TERB, this study employs fsQCA. The first step is to calibrate the data against the 5%, 95%, and 50% criteria proposed by Ragin to ensure that all values were between 0 and 1^84^. The subsequent phase involved an analysis of necessary conditions and sufficient conditions. The results revealed that the consistency level of each antecedent condition for TERB was less than 0.9; hence, no necessary conditions were identified ^85^. Subsequently, a truth table was constructed to analyze the sufficient conditions. We set the consistency threshold to 0.8^86^, the PRI threshold to 0.7^87^, and the case frequency threshold 0 to 1^71^, enabling the construction and analysis of a truth table. The results of the path normalization analysis are shown in Tables 7 and 8. The results revealed an overall consistency of 0.94 for generating TERB, with each antecedent condition construct exceeding 0.9. The overall coverage was 0.69. Furthermore, the model passed the robustness test.

**Table 7:** Combinations that lead to high levels of TERB.

Casual configuration	Raw coverage	Unique coverage	Consistency	Overall solution coverage	Overall solution consistency
Ecological presence*ESI*BV	0.69	0.69	0.94	0.94	0.69

**Table 8:** Configurations for high levels of TERB.

Solutions	Ecological presence	ESI	BV	PN	Raw coverage	Consistency
1	●	●	●		0.69	0.94

●Presence of a condition blank cell = “Do not care”.

In accordance with the fsQCA analysis standards, this study identified a configuration comprising ecological presence, biospheric values, and environmental self-identity. This configuration indicates that a robust sense of ecological presence within a VRT environment, coupled with well-established biospheric values and a pronounced environmental self-identity, constitutes a sufficient condition for achieving high levels of TERB. When integrated with the results from SEM, the pronounced sense of ecological presence in VRT emerges as a crucial catalyst, enhancing the influence of biospheric values and environmental self-identity on TERB. This synergistic interaction high-lights the critical role of immersive VRT experiences in not only captivating tourists but also aligning with their environmental values and perceptions of themselves as guardians of the environment, thus promoting a setting conducive to sustainable practices.

## Discussion and implications

### Discussion

The sense of ecological presence evoked by VRT scenarios significantly affected biospheric values, personal norms, and environmental self-identity. This outcome is primarily attributed to the satisfaction and positive experiences that VR provides tourists^18^. As suggested by the CAT, pleasant experiences foster positive evaluation and emotions of visitors’ environmental protection, which is closely associated with biospheric values, environmental self-identity, and personal norms^22^. This heightened sense of ecological presence can lead to deeper personal reflection on environmental impacts and responsibilities, thereby reinforcing one's ecological values, self-identity as an environmentally responsible individual, and intrinsic motivation to act in environmentally beneficial ways.

Biospheric values and environmental self-identity significantly impact TERB. These values and identities encapsulate the attributes of particular behaviors^52,55^. More precisely, they represent the characteristics of TERB and serve as crucial predictors of pro-environmental behavior^22^. Prior research has suggested that environmental self-identity impacts pro-environmental behavior^20^. Through the activation of biospheric values, pro-environmental behavior can be fostered, with stronger values reinforcing beliefs about such behavior^21^. Therefore, the immersive and engaging nature of the VRT environment specifically amplifies the impact of biospheric values and environmental self-identity on TERB, by making the consequences of environmental actions more salient and personally relevant to tourists, thus potentially leading to more consistent and committed pro-environmental behaviors.

The ecological presence of VRT indirectly influences TERB, primarily through enhancing biospheric values and environmental self-identity, which in turn may lead to environmentally responsible behaviors. However, this study found that, in contrast to existing research, personal norms cultivated in VRT settings do not significantly predict TERB. This discrepancy may stem from the virtual context lacking real social interactions and pressures, which are critical in developing robust personal norms that drive actual environmental behavior^88^. Consequently, while VRT effectively strengthens certain motivational factors, its isolated environment does not replicate the social reinforcement necessary for personal norms to substantially impact TERB.

By elucidating the combinatorial relationship between variables, we found that the combination of ecological presence, biospheric values, and environmental self-identity is a sufficient condition for achieving high TERB. Using fsQCA allowed us to identify that high levels of TERB are not reliant on a single factor but rather emerge from a specific configuration of conditions^89^. The analysis revealed that when a strong sense of ecological presence in VRT is coupled with deeply held biospheric values and a clear environmental self-identity, these elements interact synergistically to form a robust foundation for environmentally responsible behavior. This method highlights the complexity of TERB by demonstrating that it is the unique interplay and alignment of these conditions that is critical, rather than the strength of any individual variable^90^.

### Theoretical and practical implications

This study offers a pioneering theoretical contribution by conceptualizing 'ecological presence' as a distinct facet within VRT, a sub-dimension that encapsulates tourists' immersion in ecological virtual environments. This concept broadens the scope of presence, providing a unique perspective for assessing VRT's environmental influence. Through the application of CAT, we have dissected the internalization of VRT experiences, demonstrating how ecological presence can activate biospheric values, environmental self-identity, and personal norms. By employing a parallel mediation model, we elucidated the direct and interconnected roles these factors play in enhancing TERB. This exploration captures the complex interaction between digital immersion and the multifaceted aspects of human perception related to environmental stewardship, thus progressing our comprehension of how VRT can be instrumental in promoting behaviors that protect the environment.

Local tourism departments can leverage VRT's immersive qualities to deepen tourists' environmental commitment, going beyond enhancing perceptions to actively encouraging TERB. Insights from this research indicate that a well-crafted sense of ecological presence within VRT can inspire biospheric values and strengthen environmental self-identity and personal norms. Tourism operators are thus encouraged to prioritize VR content that emphasizes environmental narratives, offering immersive experiences that drive awareness and adoption of conservation practices among visitors. This approach can transform VR into a pivotal educational tool, promoting sustainable behaviors essential for tourist destination conservation.

### Limitations and future research directions

This study's VR technology, not utilizing head-mounted displays, may not have offered the full immersive experience afforded by the latest VR advancements. This limitation suggests that future research should employ cutting-edge VR equipment to assess whether increased immersion yields stronger influences on TERB. Furthermore, to gain a more nuanced understanding of VRT's impact, subsequent studies should explore changes in visitors' perceptions over time and include a broader array of variables such as environmental innovation, cognition, and mental imagery. Addressing these aspects could uncover deeper insights into how virtual experiences translate into real-world environmental behaviors. Additionally, incorporating a diverse range of VR technologies and settings could offer a more detailed picture of VRT's potential in fostering environmentally responsible behaviors.

### Supplementary Information

Supplementary Table S1.

## Data Availability

The raw data supporting the conclusions of this article will be made available by the author Biman Lei, without undue reservation.
