# Supplementary material for: Impact of ecological presence in virtual reality tourism on enhancing tourists’ environmentally responsible behavior
Source: Sci Rep. 2024 Mar 11;14:5939. doi: 10.1038/s41598-024-56615-z (PMC10928231; doi:10.1038/s41598-024-56615-z)
Supplement: Supplementary file 1 — Supplementary Table S1. [file 41598_2024_56615_MOESM1_ESM.pdf]

Impact of Ecological Presence in Virtual Reality Tourism on Enhancing Tourists' Environmentally Responsible Behavior

Zhen Su[1,3], Biman Lei[1], Dandan Lu[2,*], Shuchen Lai[1], Xijing Zhang[1]

1Department of Tourism Management, Business School, Guangxi University, No.100 East Daxue Road, Nanning 530004, Guangxi, China

2Department of Tourism Management, Business School, Guangxi University of Finance and Economics, Nanning 530008, Guangxi, China

3Guangxi Development Strategy Institute, Nanning 530004, China

*Corresponding. Dandan Lu ludandan@gxufe.edu.cn

**Constructs**		**Measurement Items**
Ecological presence		E-pres1: When experiencing the VR, I feel like I am in the VR's ecological environment
		E-pres2: The VR experience creates a new world for me, and the ecological world suddenly disappears when I finish it
		E-pres3: When I finish the VR experience, I feel like I am coming back to the "real world" after an ecological environment journey
		E-pres4: In the VR's ecological environment experience, sometimes I forget what I am doing
Personal Norms		PN1: VRT is environmentally friendly, so I feel morally obligated to use VRT
		PN2: VRT is environmentally friendly, so I feel that is important to use VR as a part of environmental protection
		PN3: VRT is environmentally friendly because of my own values/principles, I feel that I should use VR
		PN4: VRT is environmentally friendly, and compared to other tourism methods, I tend to prefer VRT
		PN5: VRT is environmentally friendly, I feel guilty if I do not use it
Environmental Self-Identity		ESI1: Acting environmentally friendly is an important part of who I am
		ESI2: I am the type of person who acts environmentally friendly
		ESI3: I see myself as an environmentally friendly person
Biospheric Vaues		BV1: When I see the complete natural landscape in the VR experience, I realize that I need to respect the earth
		BV2: When I see the complete natural landscape in the VR experience, I realize that I can live in harmony with other species
		BV3: When I see the complete natural landscape in the VR experience, I realize the need to unite with nature
		BV4: When I see the complete natural landscape in the VR experience, I realize the need to protect natural resources
TERB	Environment-Saving Behavior	ESB1: To reduce environmental pollution, I will choose green hotels in scenic areas for accommodation
		ESB2: I will choose tourist souvenirs with simple packaging in the scenic area
		ESB3: I will save water and electricity in the scenic area
		ESB4: When traveling in scenic areas, I will choose environmentally friendly travel methods such as hiking or public transportation

	ESB5: I will bring my own cleaning products
Environment-Promoting Behavior	EPB1: In order to protect the beautiful scenery in VR, when I see behaviors that are not conducive to the scenic environment, I will report them to the management agency<br>EPB2: When I see other tourists littering, I proactively remind them to pick up the garbage<br>EPB3: I will take the initiative to protect the environment of the scenic area, such as volunteering as a publicity volunteer, actively picking up garbage<br>EPB4: When I see public property that damages the scenic area, I will proactively remind<br>EPB5: I will prompt relatives and friends in the same industry to adopt behaviors that are beneficial to the scenic area environment
Environment-Abiding Behavior	EAB1: After experiencing the beautiful scenery in VR, I will comply with the destination's scenic area travel regulations, even if I see tourists violating the regulations<br>EAB2: I will take good care of the scenery and tourist facilities in the scenic area, even if I see other phenomena of tourists damaging them<br>EAB3: I will follow the guidance of the scenic area staff, even if I change my travel plan<br>EAB4: I will abide by the rules of the scenic area and not leave any garbage in it<br>EAB5: I will abide by the rules of the scenic area and not harm the animals and plants in the scenic area

Supplementary Table S1. Constructs and adapted source of survey items.
